# Nudix hydrolase 1 is a prognostic biomarker in hepatocellular carcinoma

**DOI:** 10.18632/aging.103083

**Published:** 2020-04-27

**Authors:** Qifeng Ou, Ning Ma, Zheng Yu, Rongchang Wang, Yucheng Hou, Ziming Wang, Fan Chen, Wen Li, Jiong Bi, Jieyi Ma, Longjuan Zhang, Qiao Su, Xiaohui Huang

**Affiliations:** 1 Laboratory of General Surgery, The First Affiliated Hospital, Sun Yat-Sen University, Guangzhou 510080, China; 2 Department of Gastrointestinal Surgery and Hernia Center, Guangdong Institute of Gastroenterology, Guangdong Provincial Key Laboratory of Colorectal and Pelvic Floor Diseases, Supported by National Key Clinical Discipline, The Sixth Affiliated Hospital of Sun Yat-Sen University, Guangzhou 510000, China; 3 Department of Gastrointestinal Surgery, The First Affiliated Hospital, Guangzhou Medical University, Guangzhou 510120, China; 4 Organ Transplant Centre, The First Affiliated Hospital, Sun Yat-Sen University, Guangzhou 510080, China; 5 Department of Pancreatobiliary Surgery, The First Affiliated Hospital of Sun Yat-Sen University, Guangzhou 510080, China; 6 Animal Center, The First Affiliated Hospital, Sun Yat-Sen University, Guangzhou 510080, China; 7 Department of Orthopaedic Surgery, The First Affiliated Hospital, Sun Yat-Sen University, Guangzhou 510080, China

**Keywords:** hepatocellular carcinoma, NUDT1, biomarker, survival analysis, nomogram

## Abstract

We investigated the prognostic significance of Nudix hydrolase 1 (NUDT1) in hepatocellular carcinoma (HCC). NUDT1 mRNA and protein levels were significantly higher in HCC tissues than normal liver tissues. The level of NUDT1 expression correlated with tumor grade, stage, size, differentiation, degree of vascular invasion, overall survival (OS), and disease-free survival (DFS) in HCC patients. Multivariate analysis showed that NUDT1 expression was an independent prognostic factor for OS and DFS in HCC patients. We constructed a prognostic nomogram with NUDT1 expression, AFP levels, vascular invasion, Child–Pugh classification, age, sex, AJCC staging, and tumor differentiation as variables. This nomogram was highly accurate in predicting the 5-year OS of HCC patients (c-index= 0.709; AUC= 0.740). NUDT1 silencing in HCC cells significantly reduced their survival, colony formation, migration, and invasiveness. Gene set enrichment analysis showed that biological pathways related to cell cycle, fatty acid metabolism, bile acid and bile salt metabolism, and PLK1 signaling were associated with NUDT1, as were the gene ontology terms “DNA binding transcription activator activity,” “RNA polymerase II,” “nuclear division,” and “transmembrane transporter activity.” Our study thus demonstrates that NUDT1 is a prognostic biomarker with therapeutic potential in HCC patients.

## INTRODUCTION

Hepatocellular carcinoma (HCC) is one of the main reasons for cancer-related deaths worldwide, especially in China [1]. The mortality rate of HCC patients is high because the cancer has already progressed before diagnosis in a majority of cases [2]. Although considerable improvements have been made in diagnosis and surgical treatments surgery, the prognosis of advanced-stage HCC remains poor because of high rates of intra- or extra-hepatic metastases [3]. Hence, there is an urgent need to identify novel diagnostic and prognostic biomarkers, and therapeutic targets to improve survival rates of HCC patients. Several genes related to HCC invasiveness and metastases have been identified [4], but further studies are required to confirm their clinical significance in HCC.

Oxidative damage is a major cause of several human diseases, including cancers [5]. Elevated levels of reactive oxygen species (ROS) have been reported in several types of cancers [6]. Nudix hydrolase 1 (NUDT1) or MutT Homolog1 (MTH1) is an enzyme that protects cells from oxidative damage by hydrolyzing oxidized nucleotides such as 8-oxo-dGTP and 2-OH-dATP, thereby preventing their incorporation into genomic DNA during DNA replication or repair [7–8]. A recent study shows that radiation induces a dose-dependent increase in NUDT1 levels in adult and pediatric glioblastoma cell lines; moreover, migration and invasiveness of the glioblastoma cell lines is inhibited by TH588, a NUDT1 inhibitor [9]. High expression of NUDT1 in tumor tissues is associated with worse overall survival (OS) and progression-free survival (PFS) of lung cancer patients [10]. NUDT1 also plays an important role in the pathogenesis of adenocarcinomas of the gastric cardia [11]. Furthermore, NUDT1 mRNA levels are elevated in colorectal cancer tissues [12].

In this study, we investigated the status of NUDT1 expression in HCC tissues and cell lines. We also explored the prognostic significance of NUDT1 in HCC.

## RESULTS

### NUDT1 mRNA expression is upregulated in HCC tissues

**Figure 1:**
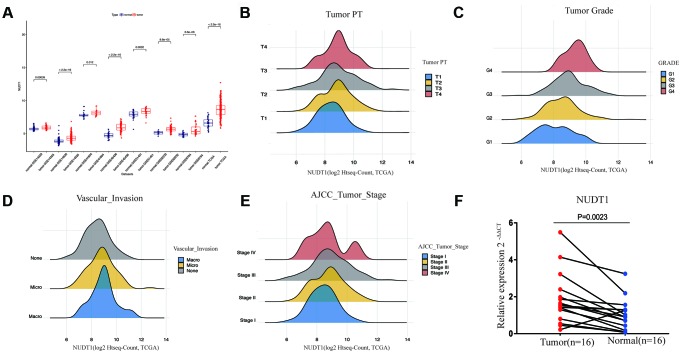
**NUDT1 is overexpressed in HCC tissues.** (**A**) NUDT1 mRNA levels in HCC and normal liver tissues in TCGA and GEO datasets. Note: TCGA dataset (tumor = 370; normal = 50); GSE14323 (tumor = 55; normal = 60); GSE14520 (tumor = 225; normal = 220); GSE41804 (tumor = 20; normal = 20); GSE45436, (tumor = 93; normal = 41); GSE51401 (tumor = 30; normal = 34); GSE62232 (tumor = 81; normal = 10); GSE6764 (tumor = 35; normal = 40). (**B**–**E**) Density plot shows the relationship between NUDT1 mRNA expression and clinicopathological characteristics, such as, tumor pathology stage (tumor PT), tumor grade, AJCC tumor stage, and degree of vascular invasion. As shown, high NUDT1 mRNA expression is associated with poor prognosis for patients with HCC belonging to stage IV, G4, macrovascular invasion, and T4. (**F**) Quantitative RT-PCR analysis of NUDT1 mRNA expression in 16 pairs of HCC and adjacent normal liver tissues is shown. NUDT1 is overexpressed in HCC tissues compared with adjacent normal liver tissues (ANLTs; P < 0.01).

NUDT1 mRNA levels are significantly upregulated in the HCC tissues from The Cancer Genome Atlas (TCGA) and the Gene Expression Omnibus (GEO) datasets when compared with the normal liver tissues (Figure 1A). Quantitative RT-PCR analysis of 16 pairs of HCC and adjacent normal tissue samples confirms that NUDT1 mRNA levels are significantly upregulated in HCC tissues compared to the adjacent normal liver tissues (Figure 1F; P = 0.0023). These data confirm that NUDT1 mRNA levels are upregulated in HCC tissues.

### NUDT1 mRNA expression is associated with tumor stage and grade

Next, we compared NUDT1 expression with clinical parameters that are related to HCC progression. As shown in Figure 1B–1E and Supplementary Table 2, NUDT1 mRNA expression positively correlates with the level of AFP expression (P = 0.000), pathological tumor stage (P = 0.038), tumor size (P=0.031), tumor grade (P = 0.000), and the degree of vascular invasion (P = 0.005). Moreover, as shown in Supplementary Table 2, a higher proportion of patients with high NUDT1 expression group show elevated AFP expression (≥ 200 ng/ml; 40% vs.), stage III-IV tumor stages (53% vs.), T3-T4 tumor size (56% vs.), G3-G4 tumor grades (47% vs.), and a higher degree of vascular invasion (42% vs.)than the patients with low NUDT1 expression. These data show that NUDT1 expression correlates with the tumor stage and grade in HCC patients.

### NUDT1 protein expression correlates with TNM stage, tumor size, and tumor differentiation in HCC

**Figure 2:**
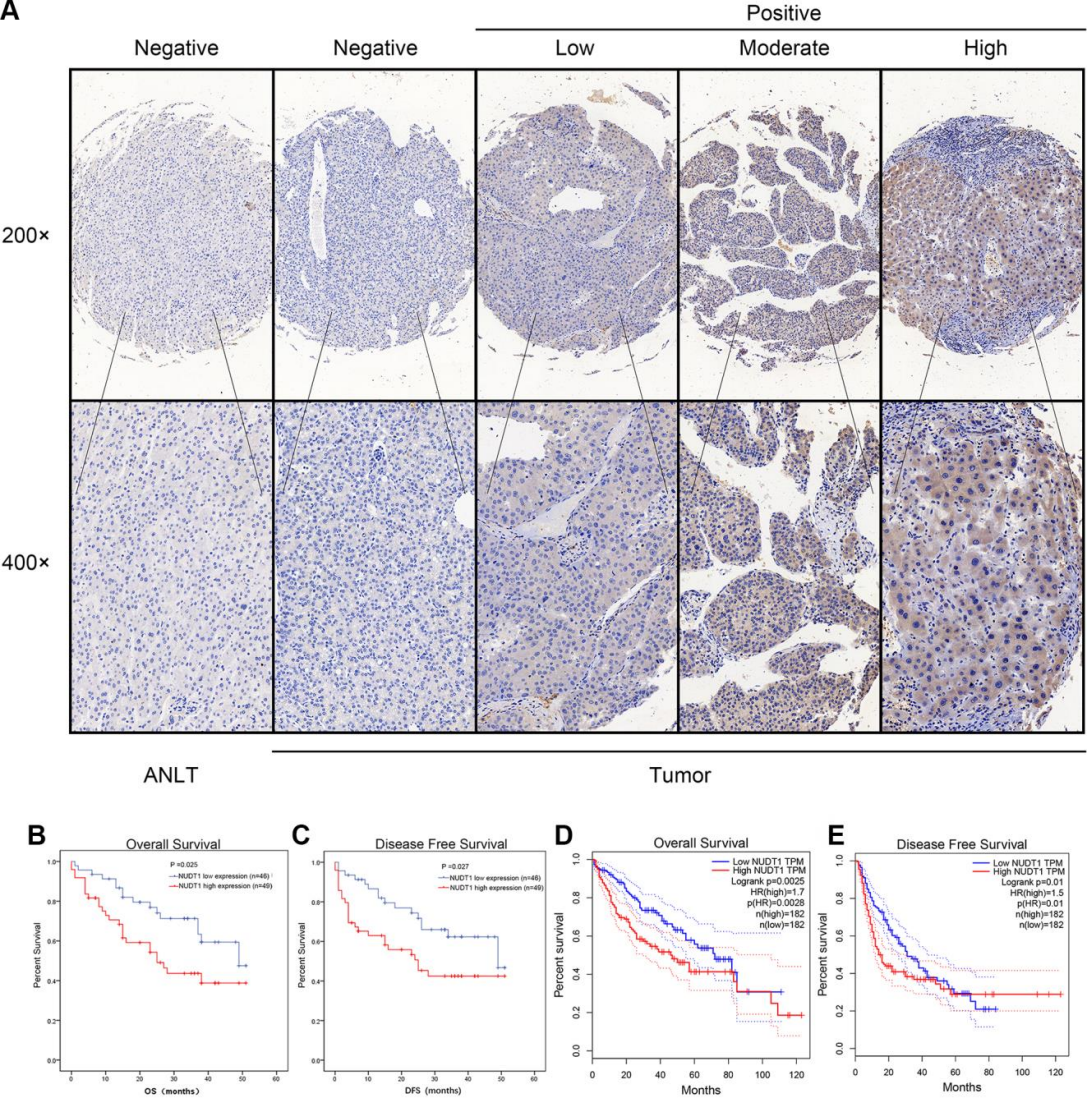
**NUDT1 protein is overexpressed in HCC tissues.** (**A**) Representative images show immunohistochemical staining results in 95 pairs of HCC and normal liver tissues samples. Normal liver tissues show negative NUDT1 expression, whereas, HCC tissues show negative or low, moderate, and high NUDT1 protein expression as shown. The scale bars indicate 50μm (200x) and 20 μm (400x). (**B**, **C**) Kaplan-Meier survival curves show overall survival (OS) and disease-free survival (DFS) curves for HCC patients with high (n=49) and low (n-46) levels of NUDT1 protein expression. (**D**, **E**) Kaplan-Meier survival curves show overall survival (OS) and disease-free survival (DFS) curves for HCC patients from the GEPIA database.

Next, we analyzed the association between NUDT1 and the clinicopathological characteristics of HCC patients. Immunohistochemical analysis showed that 49 out of 95 (51.6%) HCC patients exhibited high NUDT1 protein expression in HCC tissues compared to the adjacent normal liver tissues (Figure 2A). As shown in Table 1, NUDT1 protein expression positively correlated with the TNM stage (P =0.024), tumor size (P = 0.040), and tumor differentiation (P=0.031).

**Table 1:** Correlation between NUDT1 protein expression with clinicopathological characteristics of HCC.

**Clinical factors**	**NUDT1 level**		**Total**	***P-value***
**Group**	**High(49)**	**Low(46)**		
**Gender**				
Male	43	38	81	0.568
Female	6	8	14	
**Age (years)**				
<50	26	22	48	0.683
≥50	23	24	47	
**TNM stage**				
Early (I-II)	32	19	51	0.024*
Late (III-IV)	17	27	44	
**Cancer embolus**				
Absent	32	33	65	0.517
Present	17	13	30	
**Hepatitis virus infection**				
Negative	22	19	41	0.836
Positive	27	27	54	
**Tumor nodule number**				
Solitary	32	26	58	0.407
Multiple (≥2)	17	20	37	
**Tumor size (cm)**				
<5	20	29	49	0.040*
≥5	29	17	46	
**AFP (μg/L)**				
Low (<200)	24	26	50	0.539
High (≥200)	25	20	45	
**Differentiation grade**				
Well	28	36	64	0.031*
Poor	21	10	31	

Abbreviations: AFP, alpha-fetoprotein determination; TNM, tumor node metastasis; HR, hazard ratio; CI, confidence interval.

*Statistical significance.

### High NUDT1 expression indicates worse prognosis in HCC patients

We performed Kaplan-Meier analysis to determine the prognostic value of NUDT1 expression in HCC patients. HCC patients with high NUDT1 expression show worse overall survival (OS) and disease-free survival (DFS) compared to those with low NUDT1 expression (Figure 2B–2E). Multivariate Cox regression analysis shows that NUDT1 expression (hazard ratio (HR): 1.928; 95% confidence interval (95% CI): 1.011-3.678; P = 0.046) and TNM stages (HR: 2.179; 95% CI:1.012-4.691; P = 0.047) were independent prognostic factors for overall survival (Table 2), and NUDT1 expression (HR: 1.919; 95% CI: 1.006-3.661; P = 0.048) was an independent prognostic factor for disease-free survival (Table 3). These data demonstrate that high NUDT1 expression indicates worse prognosis in HCC patients.

**Table 2:** Univariate and multivariate Cox regression analyses of risk factors associated with overall survival.

**Variables**	**Univariate analysis**			**Multivariate analysis**		
	**HR**	**95%CI**	**P value**	**HR**	**95%CI**	**P value**
**Sex** (Male vs. Female)	0.345	0.106-1.122	0.077			
**Age** (≥50 vs. <50)	1.093	0.596-2.005	0.773			
**TNMstage** (Late vs. Early)	2.763	1.465-5.209	0.002*	2.179	1.012-4.691	0.047*
**Cancer embolus** (Presence vs. Absence)	2.340	1.260-4.344	0.007*	1.280	0.636-2.578	0.489
**Hepatitis virus infection**	1.436	0.763-2.701	0.262			
**Tumor nodule number** (Multiple vs. Single)	1.500	0.814-2.763	0.193			
**Tumor size** (≥5)	1.987	1.056-3.739	0.033*	1.250	0.622-2.514	0.531
**AFP**(≥200 ng/mL vs. <200ng/mL)	1.541	0.838-2.833	0.164			
**Differentiation grade** (Poor vs. Well)	1.879	1.023-3.453	0.042*	1.177	0.604-2.295	0.632
**NUDT1 expression** (High vs. Low)	1.999	1.070-3.737	0.030*	1.928	1.011-3.678	0.046*

Abbreviations: AFP, alpha-fetoprotein determination; TNM, tumor node metastasis; HR, hazard ratio; CI, confidence interval.

· Statistical significance.

**Table 3:** Univariate and multivariate Cox regression analyses of risk factors associated with disease-free survival.

**Variables**	**Univariate analysis**			**Multivariate analysis**		
	**HR**	**95%CI**	**P value**	**HR**	**95%CI**	**P value**
**Sex** (Male vs. Female)	0.348	0.107-1.133	0.080			
**Age** (≥50 vs. <50)	1.100	0.599-2.019	0.758			
**TNMstage** (Late vs. Early)	2.725	1.445-5.141	0.002*	2.112	0.961-4.643	0.063
**Cancer embolus** (Presence vs. Absence)	2.325	1.250-4.325	0.008*	1.254	0.615-2.558	0.533
**Hepatitis virus infection**	1.495	0.795-2.881	0.212			
**Tumor nodule number** (Multiple vs. Single)	1.578	0.853-2.918	0.146			
**Tumor size** (≥52)	2.034	1.082-3.826	0.028*	1.353	0.675-2.713	0.395
**AFP** (≥200 ng/mL vs. <200ng/mL)	1.611	0.873-2.975	0.127			
**Differentiation grade** (Poor vs. Well)	1.853	1.009-3.403	0.047*	1.108	0.562-2.181	0.768
**NUDT1 expression** (High vs. Low)	1.975	1.057-3.689	0.033*	1.919	1.006-3.661	0.048*

Abbreviations: AFP, alpha-fetoprotein determination; TNM, tumor node metastasis; HR, hazard ratio; CI, confidence interval.

*Statistical significance.

### Prognostic nomogram with NUDT1 expression as a variable

**Figure 3:**
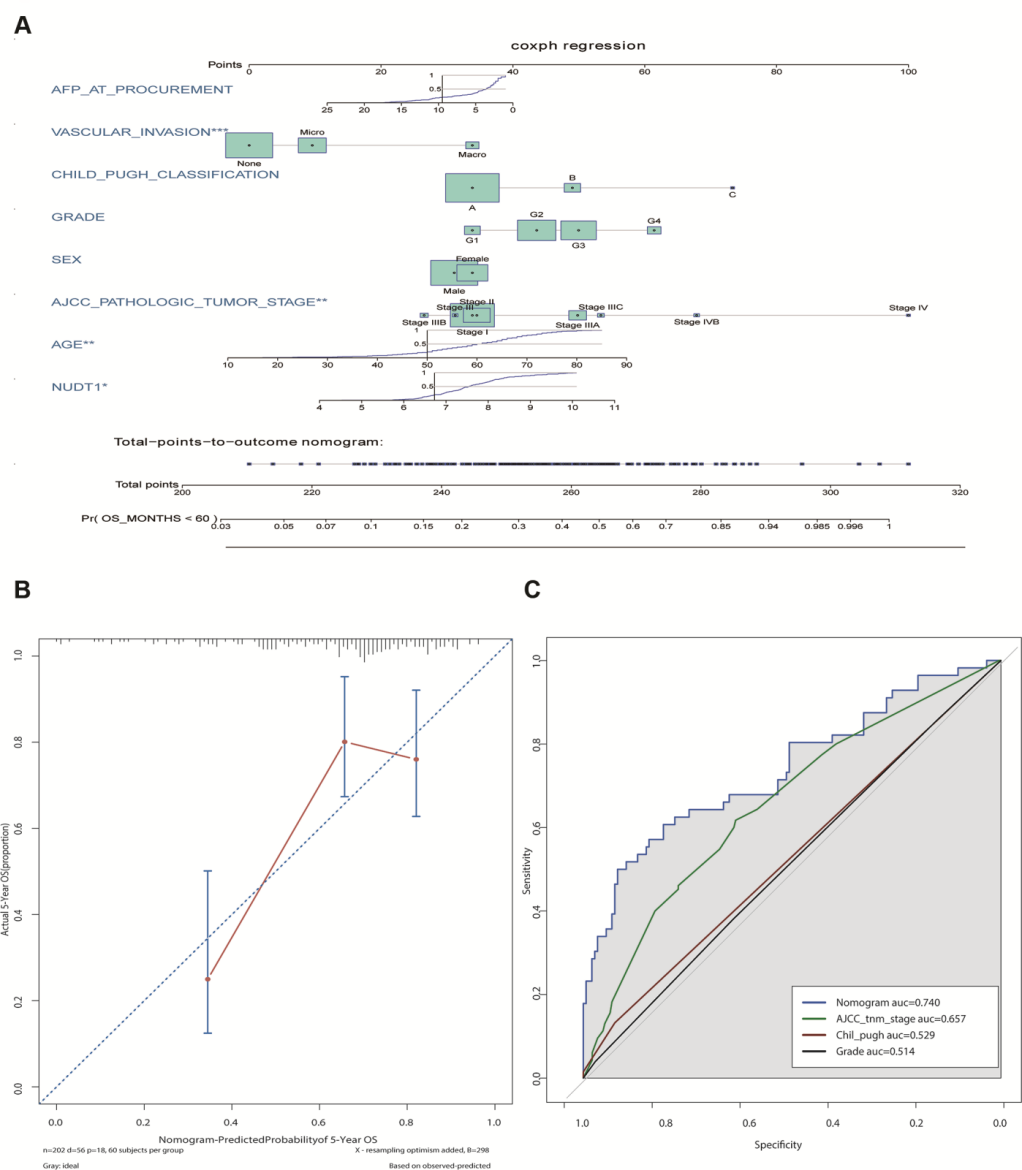
**Construction and calibration of the new prognostic nomogram to estimate 5-year survival of HCC patients using NUDT1 as a novel liver-specific variable.** (**A**) Details of the nomogram for predicting survival rates of HCC patients. The corresponding risk score of each clinical variable included in the nomogram is as listed. NUDT1 is the novel liver-specific variable in this nomogram. The C-index of the nomogram was 0.709. (**B**) The calibration plot shows the differences between true and predicted values of 5-year OS. (**C**) ROC curve analysis shows the accuracy of the novel prognostic model and other prognostic parameters. The area under curve (AUC) for the prognostic model, AJCC tumor stage, Child-Pugh classification, and tumor grade are 0.740, 0.657, 0.529, and 0.514, respectively. This shows that the prognostic model is more accurate in predicting overall survival by including NUDT1 expression as one of the parameters.

We constructed a prognostic nomogram including NUDT1 expression, and clinical factors, such as, AFP levels, vascular invasion, Child–Pugh classification, age, sex, AJCC staging, and tumor differentiation (Figure 3A). The calibration curves showed that the predictive performance of the new prognostic model was excellent (Figure 3B). The addition of NUDT1 expression as a variable improved the model accuracy in predicting prognosis. The expression of NUDT1 positively correlated with the risk score. The c-index of the new prognostic model was 0.709 (range: 0.674–0.744). Univariate Cox hazards analysis showed that AJCC stages, tumor size, tumor metastasis, and AFP levels were associated with the 5-year OS rate (Supplementary Table 3). Furthermore, ROC curve analysis showed that the new nomogram (AUC=0.740) was more accurate in predicting OS than the conventional clinical factors such as AJCC tumor stage (AUC=0.657), Child-Pugh classification (AUC=0.529), and tumor grade or stage (AUC=0.514; Figure 3C).

### NUDT1 silencing inhibits the proliferation, invasion and migration of HCC cells

**Figure 4:**
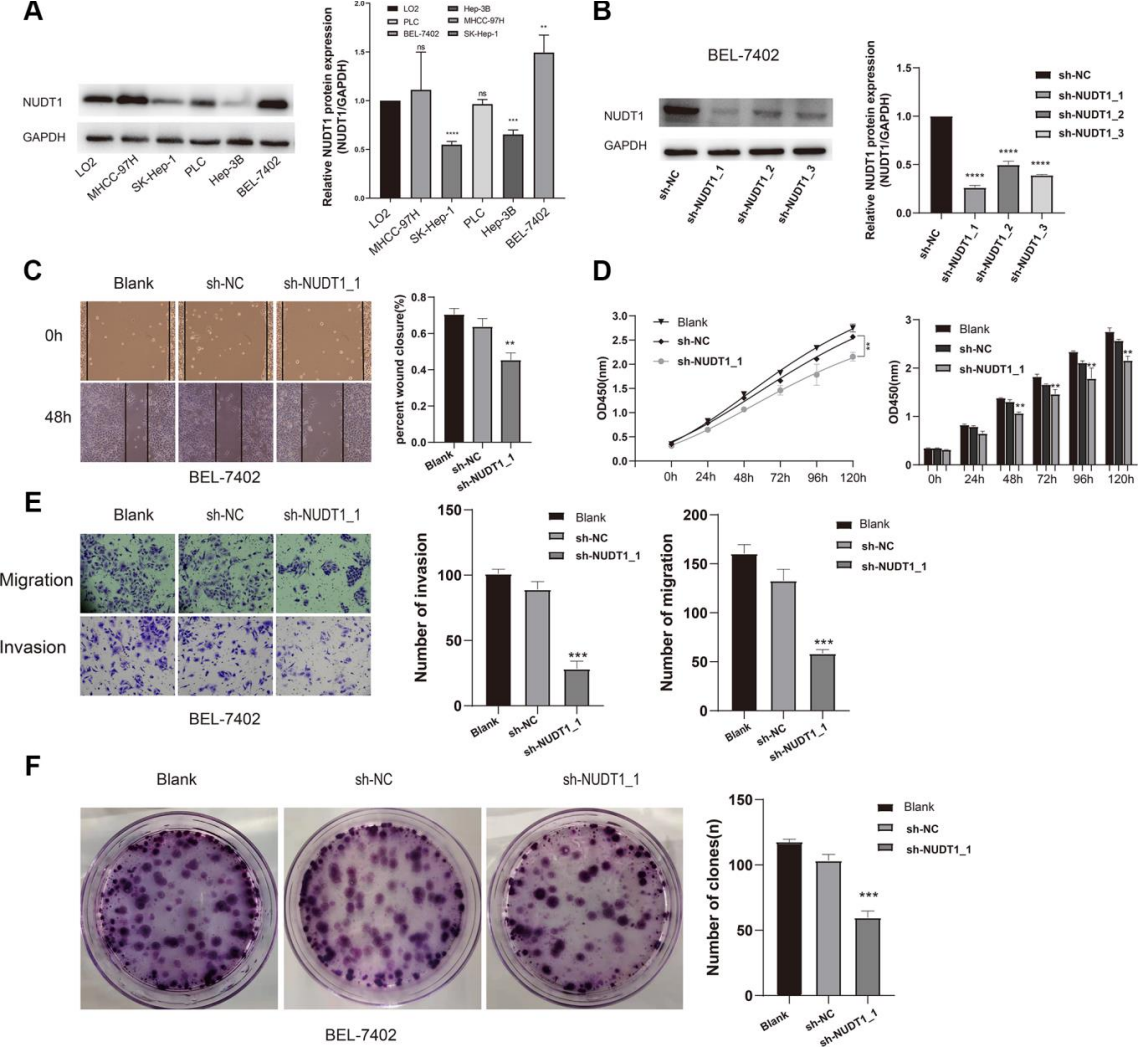
**NUDT1 silencing decreases survival, migration, and invasion of HCC cells.** (**A**) Western blot analysis shows NUDT1 expression in HCC cell lines, Hep-3B, SK-Hep-1, PLC, BEL-7402 and MHCC-97H, and the hepatic immortalized cell line, LO2. The relative levels of endogenous NUDT1 expression are shown in the right panel. (**B**) Western blot analysis shows NUDT1 protein levels in BEL-7402 cells that are transfected with three NUDT1-specific shRNAs (sh-NUDT1_1, sh-NUDT1_2, and sh-NUDT1_3) and negative control shRNA (sh-NC). The relative levels of NUDT1 protein are shown in the right panel. (**C**) Representative images show the wound healing assay results in control and NUDT1-silenced BEL-7402 at 0 and 48 h after scratching (Scale bars:100μm). The results show that NUDT1 silencing inhibits migration of HCC cells. (**D**) CCK8 assay results show that NUDT1 silencing decreases the viability of BEL-7402 cells compared to controls. (**E**) Representative images show Transwell migration and invasion assay results of control and NUDT-silenced BEL-7402 cells (Scale bars: 100μm). As shown NUDT1 silencing decreases the invasion and migration of BEL-7402 cells. (**F**) Colony formation assay results show the total number of colonies in control and NUDT1-silenced BEL-7402 cells. As shown, NUDT1 silencing decreases the colony formation in BEL-7402 cells. All the values are shown as mean ±SD of three independent experiments. Note: *** denotes *P*<0.001 as evaluated by the Student’s t-test.

To further analyze the role of NUDT1 in HCC tumorigenesis, we examined the NUDT1 protein levels in five HCC cell lines (MHCC-97H, SK-Hep-1, PLC, Hep-3B, and BEL-7402) and the normal hepatic cell line, LO2. Western blotting analysis showed that NUDT1 protein levels were significantly higher in the highly invasive human HCC cell line, BEL-7402, when compared with the normal hepatic cell line, LO2 and other HCC cell lines (Figure 4A). Therefore, we chose the BEL-4702 cell line for further analysis. We used three different NUDT1-specific shRNAs (sh-NUDT1_1, sh-NUDT1_2, and sh-NUDT1_3) to knockdown NUDT1 levels in BEL-7402 cells. We observed a 75% reduction in NUDT1 protein levels in the shNUDT1-transfected BEL-7402 cells compared to the shNC-transfected BEL-7402 cells (Figure 4B). And then we selected sh-NUDT1_1 for the subsequent experiment. CCK8 assay showed that NUDT1 knockdown significantly reduced the survival of BEL-7402 cells compared with the shNC-transfected BEL-7402 cells (P<0.001, Figure 4D). Moreover, NUDT1 silencing significantly reduced the number and the size of the colonies compared with the controls (P<0.001, Figure 2F). Wound healing assay showed delayed primary wound closure in the NUDT1-silenced BEL-7402 cells compared to the shNC-transfected BY-7402 cells, thereby suggesting reduced migration (P<0.001, Figure 4C). Transwell assay showed significantly reduced migration and invasion of NUDT1-silenced BEL-7402 cells compared to the shNC-transfected BY-7402 cells (P<0.001, Figure 4E).These results demonstrate that NUDT1 expression regulates survival, migration, and invasiveness of HCC cells.

### NUDT1-related biological pathways in HCC cells

**Figure 5:**
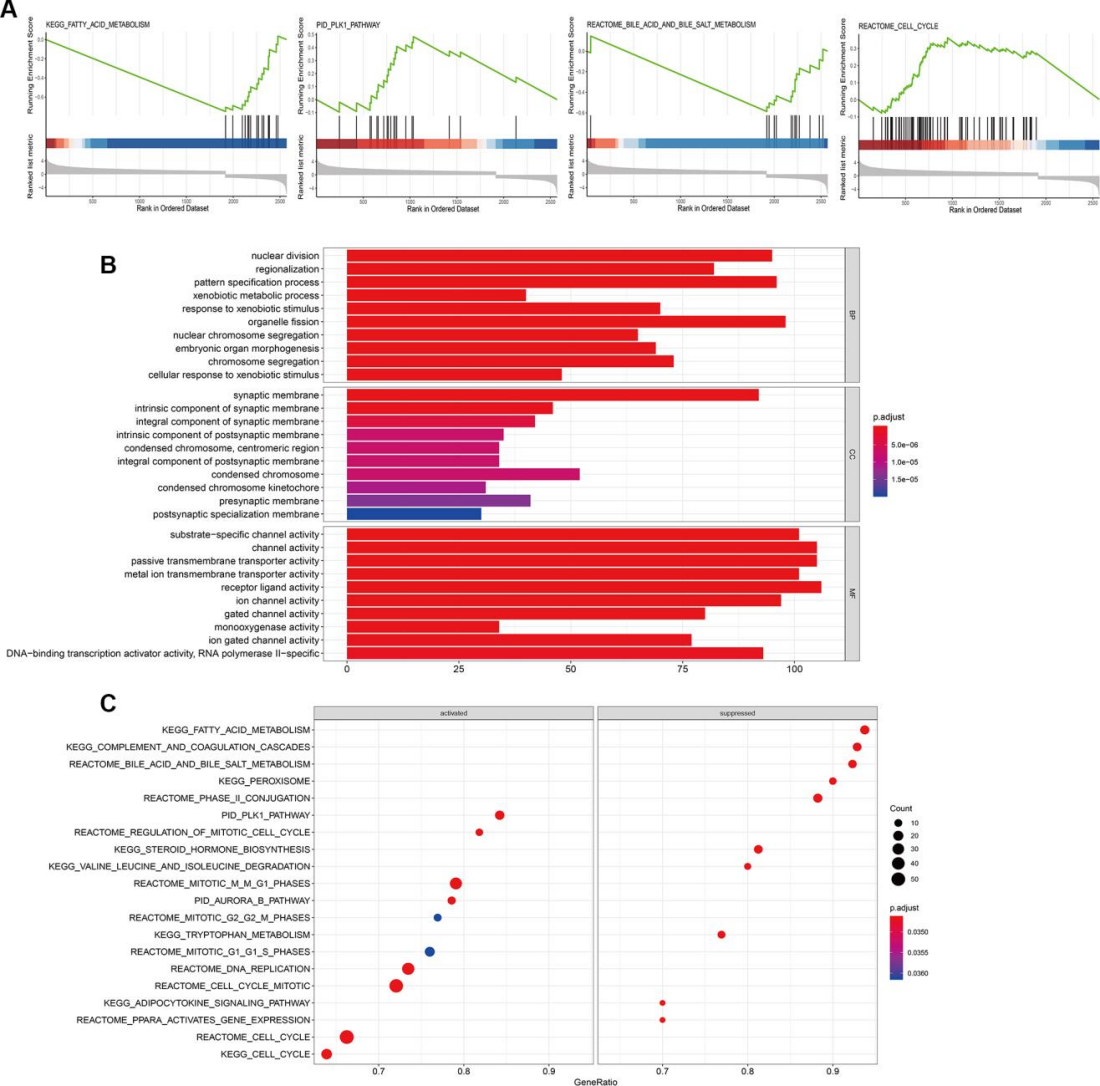
**Biological pathways regulated by NUDT1 in HCC cells.** (**A**) GSEA plot shows the key biological pathways regulated by NUDT1 in HCC cells, including fatty acid metabolism (P= 0.0034), cell cycle (P= 0.00112), bile acid and bile salt metabolism (P= 0.00326), and PLK1 pathway (P= 0.00554). (**B**) The Gene Ontology (GO) enrichment analysis plot shows the biological processes (BP), molecular functions (MF), and cellular components (CC) regulated by NUDT1 in HCC cells. (**C**) GSEA plot shows enriched signaling and cellular pathways regulated by NUDT1 in HCC cells.

We performed gene set enrichment analysis (GSEA) to identify biological pathways regulated by NUDT1 in HCC. Biological pathways related to fatty acid metabolism (P= 0.0034), cell cycle (P= 0.00112), bile acid and bile salt metabolism (P= 0.00326), and PLK1 signaling pathway (P= 0.00554) were enriched in the HCC cells with NUDT1 overexpression (Figure 5A, Supplementary Table 4). Previous studies show that pathways regulating cell cycle are aberrantly regulated in chronic hepatitis and HCC [25]. Moreover, PLK1 signaling pathway has previously been implicated in HCC invasion and metastasis [26]. In addition to these pathways, several other pathways were related to NUDT1 and are shown in Figure 5C (Supplementary Table 4). Furthermore, gene ontology (GO) terms related to DNA binding transcription activator activity, RNA polymerase II (P<0.0001), nuclear division (P<0.0001), and transmembrane transporter activity (P<0.0001) were significantly associated with NUDT1 (Figure 5B and Supplementary Table 5).

## DISCUSSION

Several HCC-related prognostic biomarkers have been identified in recent years, but most of these biomarkers are not specific to the liver tissue, are affected by several factors, and do not have therapeutic signifciance. [13] In this study, we systematically analyzed the prognostic significance of NUDT1 in HCC patients. A previous study showed that high NUDT1 protein expression was associated with poor prognosis in HCC patients [14]. In patients with chronic hepatitis C virus infection, 8-hydroxy-2-deoxyguanosine levels indicate the extent of oxidative damage to the genomic DNA, and markedly increase the risk to develop HCC [16]. Furthermore, hepatitis B virus (HBV) X protein increases the levels of 8-hydroxy-2-deoxyguanosine in the hepatocytes by inhibiting NUDT1 expression [15]. However, the clinical significance of elevated NUDT1 protein levels in HCC is not clear. In the present study, we show that high NUDT1 expression strongly correlates with advanced primary tumor, tumor grade, degree of vascular invasion, and AJCC tumor stage. The DFS and OS rates are poorer in patients with high NUDT1 expression than in patients with low NUDT1 expression. To our best knowledge, this is the first study that demonstrates the association between NUDT1 expression and clinicopathological characteristics of HCC in a relatively large number of patients. Multivariate analysis suggests that NUDT1 expression is an independent predictor of survival in HCC patients. Moreover, lack of statistical power may be the reason for the absence of correlation between several other clinicopathological factors and NUDT1 expression.

Oxidative stress plays an important role in the pathogenesis of HBV-related chronic liver diseases including HCC [17]. NUDT1 sanitizes oxidized dNTP pools and prevents incorporation of damaged oxidized bases during DNA replication [8, 18, 19]. NUDT1 overexpression has been documented in several cancers [20–23], including renal-cell carcinomas [24], brain tumors [25, 26], lung cancer [20, 27], gastric cancer [28], and esophageal squamous cell carcinomas [29]. A previous study shows that NUDT1 influences growth and survival of HCC cell lines [14]. However, the role of NUDT1 in HCC metastasis and invasion is not known. In the present study, we demonstrate that NUDT1 knockdown decreases proliferation (Figure 4D) and colony formation (Figure 4F) of HCC cell lines. Moreover, NUDT1 knockdown delays primary wound closure in the wound healing assay (Figure 4C), suggesting reduced migration of HCC cells. Furthermore, NUDT1 knockdown decreases migration and invasiveness of HCC cells in the Transwell assays (Figure 4E). These data suggest that NUDT1 promotes motility of HCC cells. GSEA analysis shows that several tumorigenesis-related pathways such as fatty acid metabolism, cell cycle, bile acid and bile salt metabolism, and PLK1 signaling pathway are regulated by NUDT1. This suggests that NUDT1 regulates cell division, proliferation, and migration, which are critical for tumor recurrence and clinical outcomes. The GO terms associated with NUDT1 in HCC cells include DNA binding transcription activator activity, RNA polymerase II, nuclear division, and transmembrane transporter activity. However, further investigations are necessary to determine the relationship between these biological pathways and NUDT1 in HCC.

Several nomograms with prognostically relevant clinicopathological variables have been used to estimate survival outcomes and the risk of early recurrence in HCC patients that have undergone hepatectomy. The tumor mutation burden (TMB) nomogram model includes tumor mutation burden, tumor size, and microvascular invasion (MVI) as variables to estimate the risk of recurrence in HCC patients [30]. Huang et al constructed a prediction model for HCC patients that included serum laminin (LN) levels, tumor size, serum AFP levels, MVI, tumor differentiation, and the number of tumors [31]. Although the variables in different prognostic prediction models are different, the C-indices are similar. The c-index of our nomogram was 0.709, which was similar to 0.65 and 0.71 for the c-indices of OS-predicting nomograms reported by Dong et al [32] and Li et al [33], respectively. We constructed a NUDT1-related nomogram using Cox proportional hazards regression analysis to determine the 5-year survival rates of HCC patients. In our model, NUDT1 gene expression is one of the variables and significantly increased the accuracy of the prognostic prediction model.

The present study has several limitations. First, the *in vivo* effects of silencing NUDT1 have not been determined. Secondly, the prediction model was not validated using third-party data and the number of clinicopathological characteristics included as variables were few. Thirdly, we did not examine serum NUDT1 levels and use them as a variable. It is plausible that the model maybe more useful if serum NUDT1 levels are used as a variable. Finally, further in-depth analysis is required to determine the role of NUDT1 and NUDT1-related proteins in HCC progression and analyze their potential as anticancer targets.

In conclusion, our study demonstrates that NUDT1 overexpression in HCC tissues indicates increased risk of recurrence and worse survival outcomes. Moreover, NUDT1 promotes proliferation, survival, migration and invasion of HCC cells. Finally, we constructed a nomogram using NUDT1 expression as one of the variables, and demonstrated improved accuracy in predicting recurrence and survival outcomes in HCC patients.

## MATERIALS AND METHODS

### Gene chip data

The RNA-seq data of HCC patients from The Cancer Genome Atlas (TCGA, http://gdc.cancer.gov/) and Gene Expression Omnibus (GEO, http://www.ncbi.nlm.nih.gov/geo) databases was analyzed to determine the relationship between NUDT1 expression in HCC patients and the clinical data obtained from the cBioPortal (http://www.cbioportal.org/). The clinical data included the 7^th^ American Joint Committee on Cancer (AJCC) stages, serum α-fetoprotein (AFP) levels, clinicopathological characteristics and the follow-up data. We obtained gene expression profiles of HCC (n = 370) and adjacent normal liver tissues (ANLT; n=50) from the TCGA database. The GEO datasets analyzed included accession numbers, GSE14323 (HCC, n = 55; normal, n =60), GSE14520 (HCC, n =225; normal, n =220), GSE51401 (HCC, n = 30; normal, n=34), GSE41804 (HCC, n = 20; normal, n=20), GSE45436 (HCC, n = 95; normal, n=39), GSE62232 (HCC, n = 81; normal, n=10) and GSE6764 (HCC, n = 35; normal, n=40).

### HCC and normal liver tissue specimens

We collected 95 paired HCC and adjacent normal liver tissue specimens that were formalin-fixed and paraffin-embedded from patients who underwent hepatic resection between July 2013 and December 2014 at the First Affiliated Hospital of Sun Yat-Sen University. The diagnoses of all patients were confirmed by pathology and none of these patients were treated with radiotherapy or chemotherapy before hepatectomy. This study was approved by the institutional review board of the First Affiliated Hospital of Sun Yat-Sen University. We obtained written consent from all patients for this study.

### Immunohistochemical staining

The tissue specimens from HCC patients were fixed in formaldehyde, paraffin embedded, and cut into 5-μm thick sections. Then, the slides were baked at 65°C for 2 h, deparaffinized, and rehydrated by incubating in serial concentrations of ethanol. Then, the specimens were pressure cooked in 10 mmol/L Tris-citrate buffer (pH 7.0) for antigen retrieval. The tissue sections were then treated with 3% hydrogen peroxide for 10 min at room temperature to block endogenous peroxidase activity, followed by incubation in 5% normal goat serum for 20 min at room temperature to block nonspecific binding of the primary antibody. The specimens were then incubated overnight at 4°C with primary anti-NUDT1 antibody (1:200 dilution; ab200832, Abcam, USA). Then, after washing in the buffer, the sections were incubated with the secondary antibody for 30 min at room temperature. The slides were developed with 3,3'-diaminobenzidine tetrahydrochloride (DAB) solution (K5007, Dako, Carpinteria, CA, USA), counterstained with haematoxylin, and photographed at 400× magnification using an Olympus BX63 microscope (Olympus, Japan). The images were quantified using the ImageJ software (National Institutes of Health, USA) and the percentages of NUDT1^+^ cells in the HCC samples. Two pathologists independently assessed and scored the specimens. The staining intensity was scored as 0, 1, 2, or 3 for negative, weak, moderate or strong, respectively. The percentage of NUDT1-positive cells were scored as 0 (absent) for < 5% positively stained cells, 1 (focal) for 5-25% positive staining, 2 (diffuse) for 25-50% positive staining, and 3 (diffuse) for ≥ 50% positive staining. The sum of staining intensity and NUDT1-positive staining scores was used to determine NUDT1 expression levels. A score of 0 or 1 indicates low NUDT1 expression, whereas higher scores indicate high NUDT1 expression.

### Real-time quantitative polymerase chain reaction (RT-qPCR)

Total RNA was extracted using the RNAiso Plus kit (TaKaRa, Japan) according to the manufacturer’s instructions and quantified using the NanoDrop 2000 instrument (Thermo Scientific, USA). The RNA samples of each pair were adjusted to the same concentration. The real-time qPCR protocol was as described previously [34]. The qPCR primers were as follows: NUDT1-F, 5’-GCTCATGGACGTGCATGTCTT-3’; NUDT1-R, 5’-GTGGAAACCAGTAGCTGTCGT-3’; GAPDH-F, 5’-GGAGCGAGATCCCTCCAAAAT-3’; and GAPDH-R, 5’-GGCTGTTGTCATACTTCTCATGG-3’.

### Prognostic nomogram with NUDT1 expression as a variable

We constructed a nomogram to predict 5-year OS using multivariate Cox proportional hazard regression analysis. The TCGA dataset consisting 372 HCC patients was used to validate the nomogram. We included NUDT1 expression (log2 transformed, Htseq-counts) as a novel variable in this nomogram. The other variables included several survival-related indicators such as age, sex, AJCC staging indicators, status of tumor differentiation, vascular invasion, and Child–Pugh classification. The C-index was used to predict the 5-year OS by the nomogram. We used the bootstrap resampling method, where we relatively selected 1000 bootstrap samples and tested the robustness using the Cox proportional hazards model. We also used area under the ROC curve (AUC) to test the accuracy of the 5-year OS prediction by the nomogram. We performed univariate and multivariate COX proportional hazards tests and the receiver operating characteristic (ROC) curve analysis to determine the performance of clinical factors as independent prognostic factors.

### Cell lines and cell culture

The human HCC cell lines, Hep-3B, SK-Hep-1, BEL-7402, and PLC, and the immortalized normal hepatic cell line, LO2 were obtained from the Cell Bank of the Chinese Academy of Sciences (Shanghai, China). The human HCC cell line, MHCC-97H, was obtained from the Liver Cancer Institute at Zhongshan Hospital of Fudan University (Shanghai, China). The cell lines were grown in Dulbecco’s modified Eagle’s medium (DMEM, Gibco, USA) supplemented with 10% fetal bovine serum (FBS, Gibco, USA) and 1% penicillin/streptomycin in a humidified air chamber at 37 °C and 5% CO_2_.

### Plasmid transfection

The NUDT1-specific shRNA plasmids were obtained from GeneCopoeia (Guangzhou, China). The target sequences are listed in Supplementary Table 1. The empty vector was used as a negative control. We performed transient transfections of BEL-7402 cell line with control and NUDT1-specific shRNAs using the Neofect™ reagent (Neofect Biotechnologies, Guangzhou, China) according to the manufacturer's instructions. Briefly, 1.5 × 10^5^ BEL-7402 cells were seeded per well in 6-well culture plates and grown until they obtained 70% confluence. Then, the cells in 200 μL Opti-MEM medium were transfected with 2.0 μg control or NUDT1-specific shRNAs in 2 μL Neofect™ reagent. Then, 24 h after transfection, the medium was changed to DMEM medium supplemented with 10% fetal bovine serum (FBS, Gibco, USA) and 1% penicillin/streptomycin. The transfection efficiency was estimated by qRT-PCR and western blotting analysis of NUDT1 mRNA and protein levels, respectively. The efficiency of transfection was ~70% for all the experimental groups.

### Transwell migration and invasion assays

The HCC cell migration and invasion assays were performed using 8 μm pore size Transwell chambers (Corning, NY, USA), with or without Matrigel (Matrigel in DMEM or RPMI 1640 medium in a 1:8 ratio; 50 μL per well; BD Biosciences, NJ, USA). Briefly, 5 × 10^4^ cells were seeded per well in the upper chambers of the Transwell in 200 μL of serum-free medium for the migration assay, whereas, 3 × 10^4^ cells were seeded per well in 200 μL of serum-free medium in the upper chambers of the Transwell that was coated with matrigel for the invasion assay. In the bottom chambers, we added 700 μL of medium containing 20% FBS as a chemoattractant. Then, the Transwell chambers with cells were incubated at 37 °C for 24 or 48 h. Then, the cells on the upper chamber side of the Transwell were removed with a cotton swab. The cells that migrated or invaded the lower surface of the filter were fixed in 4% paraformaldehyde, stained with crystal violet, and counted at 100× magnification in five random fields under a light microscope. All the experiments were performed in triplicates.

### CCK-8 cell viability assay

We used the Cell Counting Kit-8 (CCK-8) (Dojindo, Japan) to estimate viability of cells. The transfected cells were seeded into 96-well plates at a concentration of 2 × 10^3^ cells per well in 100 μL complete medium and cultured at 37°C and 5% CO_2_ for 24 h. Then, we incubated the cells for another 2 h after adding 100 μL of CCK-8 solution into each well. We measured the absorbance at 450 nm using a micro-plate reader.

### Colony formation assay

Briefly, 300 cells per well from exponentially growing cell cultures were seeded into 3.5 cm cell culture dishes and cultured at 37°C and 5% CO_2_ for 10–15 days. The colonies were fixed in 75% ethanol for 30 mins, stained with 0.1% crystal violet, and visualized under a light microscope. The colonies with more than 50 cells were counted in each experimental group. Triple wells were counted for each experimental group, and all experiments were repeated thrice.

### Western blotting

Total protein lysates were prepared using the RIPA buffer and the protein concentration was quantified using the BCA Protein Assay kit (Keygentec, Nanjing, China). Equal amount (30 μg) of protein samples were separated by 10% sodium dodecyl sulfate-polyacrylamide gel electrophoresis (SDS-PAGE) and transferred onto polyvinylidene fluoride (PVDF) membranes (EMD Millipore, MA, USA). Then, the membranes were blocked in 5% bovine serum albumin (BSA) in Tris-buffered saline containing 0.1% Tween 20 (TBST) at room temperature for 1 h. The membranes were incubated overnight at 4°C with primary antibodies, namely, rabbit monoclonal anti-NUDT1 (1:1000 dilution; ab200832, Abcam, USA), and rabbit polyclonal GAPDH (1:2000; Bioss, Beijing, China). Then, after washing with TBST, the membranes were incubated for 2 hours at room temperature with the corresponding secondary HRP-conjugated antibody (1:7000; goat anti-rabbit; Cell Signaling Technology, USA). Then, the blots were washed with TBST for 10 min and developed using the Millipore Immobilon Western Chemiluminescent HRP Substrate (EMD Millipore, Billerica, MA, USA).

### Wound scratch assay

A scratch was made in a confluent monolayer of cells in a six-well plate using a sterile 100μl pipette tip. The scratch wound was imaged at 0 h and 48 h in three different fields and the area of the open wound was quantified using the Adobe Illustrator CC 2018 (Adobe, USA) to determine the extent of migration of the cells adjacent to the wound.

### GSEA and GO analyses to determine NUDT1-related biological pathways

We used “ClusterProfiler” and “DOSE” R language packages to perform pathway enrichment analysis to investigate the biological pathways that correlate with NUDT1. Briefly, the samples in the TCGA dataset were classified into high-expression and low-expression groups according to the median value of NUDT1 expression. Then, we analyzed the differentially expressed genes (DEGs) using the “DEseq2” R package with a P-value <0.05 as the threshold. Gene set enrichment analysis (GSEA) was performed to determine the biological pathways that correlate with NUDT1 using Kyoto Encyclopedia of Genes and Genomes (KEGG) and Reactome and Pathway Interaction Database (PID). Gene Ontology (GO) enrichment analysis was performed to determine the biological processes, molecular functions, and cellular components that were altered in a NUDT1-dependent manner in the HCC samples.

### Statistical analysis

Statistical analysis was performed with the R software version 3.50 (http://www.r-project.org). The differences between groups were analyzed by Student’s t or Fisher’s exact tests. The prognostic potential of clinical variables was determined using Cox regression and Kaplan-Meier analyses. Two-tailed P < 0.05 was considered statistically significant. Univariate and multivariate analyses was performed using the “survival” packages in the R software and the density plots were drawn using “ggplot2”. The “DEseq2” and “edgeR” packages were used to analyze Htseq-counts for the TCGA dataset and the RSEM (GEO, log2 scaled) and “rms” packages were used for the nomogram. The “ClusterProfiler” and “DOSE” packages were used for pathway enrichment analysis.

## Supplementary Material

Supplementary Tables

Supplementary Table 4

Supplementary Table 5
