# Supplementary material for: Nudix hydrolase 1 is a prognostic biomarker in hepatocellular carcinoma
Source: Aging (Albany NY). 2020 Apr 27;12(8):7363–79. doi: 10.18632/aging.103083 (PMC7202498; doi:10.18632/aging.103083)
Supplement: Supplementary Tables [file aging-12-103083-s002..pdf]

SUPPLEMENTARY TABLES

Supplementary Table 1. The sequences of shRNA target.

Clone name	Symbol	Target sequence
HSH011544-LVRU6GP-a (sh-NUDT1_1)	NUDT1	GGCAAAGTGCAAGAAGGAGAG
HSH011544-LVRU6GP-b (sh-NUDT1_2)	NUDT1	CGAGCCTGAGCTCATGGACGT
HSH011544-LVRU6GP-c (sh-NUDT1_3)	NUDT1	CACTCCTGCTTCAGAAGAAGA

Supplementary Table 2. Association of NUDT1 mRNA expression with clinicopathological characteristics of HCC (TCGA).

Clinical factors	NUDT1 level		Total	*P-value*
**Group**	**High(185)**	**Low(185)**		
**Gender**				
Male	120(65%)	130(70%)	250	0.318
Female	65(35%)	55(30%)	120	
**Age**				
<50	37(20%)	33(19%)	70	0.670
≥50	147(80%)	152(81%)	299	
**AFP**				
<200	81(60%)	119(84%)	120	1.424e-05*
≥200	54(40%)	23(16%)	77	
**Tumor_pathologic_stage**				
Stage I-II	118(69%)	138(79%)	256	0.038*
Stage III-IV	53(31%)	37(21%)	90	
**Tumor size**				
T1-T2	129(70%)	146(79%)	275	0.031*
T3-T4	56(30%)	37(21%)	93	
**Tumor metastasis**				
M0	138(98%)	128(99%)	266	0.623
M1	3(2%)	1(1%)	4	
**Tumor Nodes**				
N0	124(98%)	128(99%)	252	0.368
N1	3(2%)	1(1%)	4	
**Child pugh classification**				
A	103(94%)	111(87%)	214	
B	7(6V)	15(12%)	22	0.181
C	0(0%)	1(1%)	1	
**Height mean**	166.21	168.48		0.051
**Grade**				
G1	17(9%)	37(20%)	54	
G2	80(44%)	96(53%)	176	7.81E-05*
G3	76(42%)	47(26%)	123	
G4	10(5%)	2(1%)	12	
**Vascular invasion**				
Macro	11(7%)	5(3%)	16	0.005*

Micro	54(36%)	38(23%)	92	
Non_vascular_invasion	85(57%)	120(74%)	205	
**Hepatic_infalmmation_adj_tissue**				
Mild	44(41%)	54(43%)	98	
Severe	6(6%)	13(10%)	19	0.346
None	58(53%)	59(47%)	117	
**Tumor status**				
Tumor free	111(66%)	122(70%)	233	0.419
With tumor	58(34%)	52(30%)	110	

Abbreviations: AFP, alpha-fetoprotein determination.
*Statistical significance.

Supplementary Table 3. Cox proportional-hazard regression analysis for overall survival in TCGA dataset (HCC).

Variables	No.	Unvariable analysis		Multivariable analysis	
		HR (95%CI)	P	HR (95%CI)	P
**Gender**					
**F**	121	0.8851(0.6108-1.283)	0.52	0.7501(0.3693-1.523)	0.4262
**M**	247				
**Age**					
≤60	176	1.212(0.845-1.737)	0.297	1.5019(0.7576-2.977)	0.2441
**>60**	191				
**AFP(ug/L)**					
**>20**	131	1.794(1.143-2.816)	0.011	1.394(0.7190-2.703)	0.3254
≤20	146				
**Tumor AJCC stage**					
**Stage I-II**	255	2.541(1.731-3.731)	9.40E-07	1.0041(0.1040-9.698)	0.9972
**Stage III-IV**	89				
**Tumor Size**					
**T1-T2**	273	2.674(1.857-3.849)	1.23E-07	1.1398(0.1125-11.544)	0.9118
**T3-T4**	92				
**Tumor metastasis**					
**M0**	264	4.022 (1.264-12.8)	0.0185	3.9127(0.9533-16.059)	0.0583
**M1**	4				
**Tumor Nodes**					
**N0**	251	1.988(0.4872- 8.117)	0.338	NA	NA
**N1**	4				
**Child pugh classification**					
**A**	215	0.5516(0.2722-1.118)	0.0988	0.4125(0.1551-1.097)	0.076
**B-C**	22				
